# Maternal and fetal/neonatal outcomes in pregnancy, delivery and postpartum following bariatric surgery and comparison with pregnant women with obesity: a study protocol for a prospective cohort

**DOI:** 10.1186/s12978-023-01736-3

**Published:** 2024-01-17

**Authors:** Maryam Navaee, Maryam Kashanian, Ali Kabir, Negar Zamaninour, Maryam Chamari, Abdolreza Pazouki

**Affiliations:** 1 Minimally Invasive Surgery Research Center, Iran University of Medical Sciences, Tehran, Iran; 2 Department of Obstetrics and Gynecology, School of Medicine, Iran University of Medical Sciences, Tehran, Iran; 3 Department of Community Nutrition, School of Nutritional Sciences and Dietetics, Tehran University of Medical Sciences, Tehran, Iran; 4 Center of Excellence of European Branch of International Federation for Surgery of Obesity, Tehran, Iran

**Keywords:** Perinatal outcomes, Postpartum, Bariatric surgery, Obesity, Micronutrients, Stress, Anxiety, Depression

## Abstract

**Background:**

Being obese can lead to various complications during pregnancy, such as Gestational Diabetes Mellitus (GDM), pregnancy induced hypertension (PIH), Pre-Eclampsia (PE), and Large Gestational Age (LGA). Although bariatric surgery is an effective way to treat obesity, it can also result in complications and may be linked to having small for gestational age (SGA) babies. This cohort study protocol aims to compare the maternal and fetal/neonatal outcomes of two groups of Iranian pregnant women: those who have undergone bariatric surgery and those who are obese but have not had bariatric surgery.

**Methods:**

In this study Pregnant women (< 14 weeks’ gestation) (n = 38 per group) are recruited either from one of the obesity clinic (exposure group = with a history of bariatric surgery) or primary healthcare clinics in Tehran city (comparison group = pregnant women with obesity and and no history of bariatric surgery). Dietary intake and nutrient status are assessed at < 14, 28, and 36 weeks. Maternal and fetal/neonatal outcomes are compared between the two groups, including gestational diabetes, preeclampsia, preterm labor, intrauterine growth restriction, severe nausea and vomiting, abortion, placenta previa and abruption, venous thrombosis, vaginal bleeding, cesarean delivery, meconium aspiration, and respiratory distress. Maternal serum levels of ferritin, albumin, zinc, calcium, magnesium, selenium, copper, vitamins A, B9, B12, and 25-hydroxy Vit D are checked during 24th to 28th weeks. Maternal and neonatal outcomes, including height, weight, head circumference, fetal abnormality, infection, small or large fetus, low birth weight, macrosomia, NICU admission, and total weight gain during pregnancy, are measured at birth. Maternal and offspring outcomes, including weight, height, head circumference, total weight gain during pregnancy, newborn diseases, postpartum bleeding, breastfeeding, and related problems, are assessed 6 weeks after delivery. Child's weight, height, and head circumference are followed at 2, 4, 6, 8, 10, and 12 months after birth. Maternal stress, anxiety, and depression are assessed with the DASS-21 questionnaire, and physical activity is evaluated using the PPAQ questionnaire in the first and third trimesters.

**Discussion:**

By assessing the levels of micronutrients in the blood of pregnant women along with the evaluation of pregnancy outcomes, it is feasible to gain a more accurate understanding of how bariatric surgery affects the health and potential complications for both the mother and the fetus/newborn. This information can help specialists and patients make more informed decisions about the surgery. Additionally, by examining issues such as stress, anxiety, and depression in women undergoing surgery, this study can contribute to recognizing these problems, which can also affect pregnancy outcomes.

## Background

### Obesity rates in Iran and globally

Obesity is a chronic condition influenced by genetic, metabolic, behavioral, environmental, cultural, and psychological factors [1] and it poses a significant threat to human health in the twenty-first century. Obesity has become a significant global issue due to its rapid growth worldwide [2]. The fourth Global Obesity Atlas 2020 predicts that by 2030, one billion people worldwide will be overweight or obese, with 1 in 5 women and 1 in 7 men affected. This indicates a doubling of the current number of obese individuals globally [3]. This issue is also growing in Iran, where 20% of Iranian adult men and 34% of Iranian women are affected by this condition [4]. Therefore, It is crucial for governments worldwide to collaborate with the World Health Organization in developing a comprehensive global action plan to address this issue [3].

### Complications of obesity in pregnancy

According to the World Health Organization, more than half of Iranian women are overweight or obese [2] and one in five women experience overweight or obesity during pregnancy [5]. The mother's pre-pregnancy body mass index and pregnancy weight gain can directly affect both maternal and birth outcomes [6]. Studies conducted in different regions of Iran have shown that pregnancy weight gain and pre-pregnancy body mass index can lead to adverse pregnancy outcomes [7–9]. Women with obesity and their fetuses are at a higher risk of miscarriage, stillbirth, Preeclampsia, large fetus for gestational age, gestational diabetes, premature delivery, cesarean delivery, meconium aspiration, and respiratory distress during pregnancy and delivery [10–12]. Maternal obesity can also lead to congenital abnormalities such as neural tube defects and abnormal intrauterine growth, which can have lifelong side effects, including obesity in the growing child [13]. Additionally, obesity is an independent predictor of longer hospital stays following complicated deliveries. Therefore, it is crucial to find definitive solutions to treat obesity and its complications, especially during pregnancy [14].

### Bariatric surgery

Bariatric surgery has become increasingly consequential in controlling weight and exorbitant corpulence due to the transitory and inhibited effects of lifestyle changes and drug treatments [15]. This type of surgery causes weight loss by altering the digestive system. The most commonly used procedures for treating morbid obesity are gastric bypass (Roux-en-Y), banding, and sleeve gastrectomy. Studies have shown that the sleeve method is more effective and has fewer complications than other surgical methods for long-term weight loss maintenance [16]. Women are five times more likely than men to undergo bariatric surgery, with over 80% of patients being women between 18 and 45 years old. Bariatric surgery is the most successful and enduring method for treating obesity [17], it is associated with complications such as cardiovascular complications, deep thrombophlebitis, gallstone formation, mental disorders, deficiencies of macronutrients and micronutrients, and even death [18]. These deficiencies can occur in over 46% of patient’s even years after the operation, with anemia being seen in over 70% of patients [19]. For instance, folic acid deficiency has been reported in 38% of Roux-en-Y patients. It is especially important to treat these complications in women who plan to become pregnant to prevent nervous system disorders in the fetus [20].

### Maternal–fetal outcomes in pregnancy after bariatric surgery

Bariatric surgery is generally safe, but its risks during pregnancy and delivery are uncertain [21]. Based on the control group used, different results have been reported regarding maternal-fetal complications during pregnancy. In the comparison of pregnancies in women with a history of bariatric surgery with women having normal body mass index, a higher incidence of pregnancy complications has been observed. This is in contrast to when obese women were used as the control group, often no significant reduction in complications was reported [11, 12, 22, 23]. Bariatric surgery may reduce the occurrence of obesity-related complications during pregnancy, but not to the same extent as in the population of women with normal weight [17].

A meta-analysis showed lower rates of preeclampsia and gestational diabetes, but increased maternal anemia [24]. However, there was variation among the studies used in the meta-analysis on pregnancy-induced hypertension [23]. While most studies did not show any difference between the two groups with and without a history of surgery, one study showed a lower prevalence of hypertension in pregnant women who underwent bariatric surgery compared to pregnant women with obesity [25]. The rate of gestational diabetes after bariatric surgery may decrease due to absorption or metabolic changes [26]. Systematic reviews on gestational diabetes have shown a decrease or no change in the rate of gestational diabetes among bariatric surgery patients [27]. Other reported complications of bariatric surgery during pregnancy include intestinal obstruction or hernia and gastric ulcer, which require a quick response to minimize risks to the mother and fetus [20]. A Swedish cohort study identified a high risk of abdominal surgery and intestinal obstruction during pregnancy [28] and case and series studies have described small bowel obstruction or internal hernia during pregnancy [29].

Protein, iron, folate, calcium, and vitamin B12 and D are the most common nutrient deficiencies after gastric bypass surgery, which may also have negative effects on the fetus [30]. The need for nutrients during pregnancy increases due to fetal growth, and a deficiency in any of them may increase the risk of fetal abnormalities, especially neural tube defects [31]. Nausea and vomiting in pregnancy can be a very influential factor in exacerbating maternal nutritional deficiencies, which may have serious consequences [20]. Studies on birth weight after bariatric surgery show a decrease in average birth weight and fetal macrosomia, and an increase in small for gestational age (SGA) fetuses and intrauterine growth restriction (IUGR) of infants [11, 12, 23]. These trends are most likely caused by maternal malnutrition following bariatric surgery and micronutrient deficiency, which may be exacerbated during pregnancy [22].

### Maternal and fetal-neonatal outcomes during labor and delivery

Bariatric surgery can also affect the mother and fetus during childbirth. Some studies have reported an increase in cesarean rates in women who have undergone bariatric surgery [12, 32], but these results are contradictory [33]. Two meta-analyses on bariatric surgery and delivery outcomes reported significant differences in cesarean delivery and postpartum hemorrhage between women with and without a history of bariatric surgery [34, 35]. Stephansson et al. (2018) found that the group with a history of surgery had lower rates of emergency cesarean sections and postpartum bleeding compared to the obese group without a history of surgery [22]. In Balestrin's study, there were no significant differences in prematurity, delivery methods, or postpartum complications [25]. However, Kauken's study showed that preterm labor, labor induction, planned and unplanned cesarean sections, and low birth weight babies were more common in the obesity group [36]. In another study, Sheiner et al. reported similar rates of placental abruption, labor dystocia, and meconium-stained amniotic fluid between women who had undergone bariatric surgery and the general population [37].

### Nutritional status and levels of micronutrients in pregnancy

During pregnancy, the body's increased nutrient requirements, combined with the additional demands of surgery, can lead to deficiencies in essential micronutrients that can negatively impact both the mother and the developing fetus [38, 39]. Common deficiencies include iron, vitamin B1, vitamin B12, folic acid, vitamin D, and calcium [40]. These deficiencies can be caused by a decrease in dietary intake, reduced tolerance of certain foods, decreased gastric acid secretion, and the elimination of absorption sites [41]. Vomiting and nausea during pregnancy, as well as a lack of supplementation, can exacerbate these deficiencies over time [42]. For instance, the serum level of vitamin B1 decreases during normal pregnancy, but this decrease is more pronounced in pregnancies after bariatric surgery [43]. Hemoglobin concentration, ferritin, and average blood volume also decrease during normal pregnancy due to hemodilution, while pregnancy after bariatric surgery can lead to iron deficiency due to reduced meat consumption and intestinal iron absorption capacity [44]. Nutritional deficiencies during pregnancy after bariatric surgery can have serious consequences for the baby. Therefore, large cohort studies are needed to determine the prevalence and extent of micronutrient deficiencies in this population and to identify the optimal diets and micronutrient requirements to prevent these deficiencies [45].

### Stress, anxiety and depression in pregnant women with obesity with and without history of bariatric surgery

Mental disorders affect 10% of pregnant women worldwide [46], with stress, anxiety, and depression being the most prevalent [47]. Prevalence of prenatal depression ranges from 7 to 20% in high-income countries and 20% or higher in low- and middle-income countries [48]. In Iran, a study found that 25% of pregnant mothers reported symptoms of depression, and half of them experienced anxiety during pregnancy [49]. These disorders can negatively impact fetal growth and development, leading to an increased risk of complications such as abortion or preterm labor [47]. It's crucial to assess and treat symptoms early to reduce negative consequences for maternal and child health [50].

Factors like poverty, education, family income, mother's age, unwanted pregnancy, domestic violence, and obesity can affect pregnant women's mental health [51]. Obesity is linked to higher levels of depression, anxiety, and physical complaints [52]. In Iran, depression is 2–3 times more prevalent in obese people than those slightly overweight [53, 54]. However, some studies found no significant link between body mass index and depression in pregnant women [55].

36% seeking bariatric surgery have depression, 11% have anxiety [56]. However, most people experience a significant improvement in psycho-social functioning after the surgery [57]. Studies have shown that symptoms of depression improve in 67% of individuals within 3 to 6 months after the surgery [58]. Bariatric surgery reduces psychiatric symptoms more than non-surgical treatment. However, rapid changes may cause psychological disorders like depression, anxiety, alcoholism, and bulimia nervosa [59]. Pregnant women who had bariatric surgery are vulnerable to mental disorders during pregnancy, with depression/anxiety rates higher than those without surgery [50]. Furthermore, Kim et al. reported that in the group of pregnant women with a history of bariatric surgery, the rate of depression/anxiety was 24.4%, while this rate was reported as 14.3% inpregnant women with obesity without a history of bariatric surgery [60]. Factors like changes in intestinal microbiota and nutrient absorption may contribute to mental disorders [61, 62]. During pregnancy, this issue can be related to changes in food absorption and issues and fears related to the more difficult adaptation of these people to pregnancy (due to changes in physical and mental levels caused by bariatric surgery) [63]. Relationship between psycho-social stress, diet, and body weight change during pregnancy is inconsistent [64, 65].

### Necessity of project implementation

Currently, there is growing clinical concern in obstetrics and gynecology about the potential issues arising from bariatric surgery in pregnant and postpartum women. The combination of physiological changes related to surgery and pregnancy may increase the risk of clinical failure [66]. Additionally, adequate intake of micronutrients is crucial for the mother's mental health, and deficiencies have been linked to symptoms of depression and anxiety during and after pregnancy [67]. Therefore, it is vital to closely monitor the nutritional status of these women to ensure the well-being of both the mother and the fetus/newborn [66]. Despite the increasing number of bariatric surgeries in Iranian women and subsequent pregnancies, there is currently no cohort or comparative study in Iran investigating the outcomes of pregnancy and childbirth in women with a history of bariatric surgery. Given these circumstances, there is a need for cohort and comparative studies in this field. This report outlines the study protocol for a cohort study that evaluates maternal and fetal/neonatal outcomes during pregnancy, labor, and postpartum in two groups: pregnant women with a history of bariatric surgery and pregnant women with obesity.

### Study objectives

#### Primary objectives

1. Determining and comparing the frequency of maternal outcomes during pregnancy and after delivery in two groups with and without history of bariatric surgery.
2. Determining and comparing the frequency of fetal/neonatal outcomes during pregnancy and after delivery in two groups with and without history of bariatric surgery.
3. Determining and comparing the average score of stress, anxiety and depression of pregnant women in two groups with and without history of bariatric surgery.

#### Secondary objectives

1. Determining and comparing the levels of laboratory parameters and micronutrients in maternal serum in two groups with and without history of bariatric surgery
2. Determining and comparing the relationship between the level of maternal serum micronutrients and maternal and fetal pregnancy outcomes in two groups with and without a history of bariatric surgery.

#### Study hypotheses

1. The frequency of maternal outcomes during and after pregnancy differs between the two groups with and without a history of bariatric surgery.
2. The frequency of fetal/neonatal outcomes during and after pregnancy differs between the two groups with and without a history of bariatric surgery.
3. The levels of laboratory parameters and micronutrients in maternal serum differ between the two groups with and without a history of bariatric surgery.
4. There is a relationship between maternal serum micronutrient levels and maternal/fetal pregnancy outcomes in the two groups with and without a history of bariatric surgery.
5. The average levels of stress, anxiety, and depression in pregnant women differ between the two groups with and without a history of bariatric surgery.

#### Study design

We are undertaking a prospective cohort study tracking 136 women through pregnancy to childbirth and following their infants until they are 12 months old. These women are split into two groups (1:1 ratio): 68 pregnant women who have had bariatric surgery in the past and 68 pregnant women with obesity but no history of bariatric surgery. We are collecting data in early pregnancy, mid-pregnancy, late pregnancy, and at birth. After birth, we are focusing on maternal and neonatal complications during the postpartum period (first 6 weeks after delivery) and tracking the development and outcomes of the infants at 4th, then 2, 4, 6, 8, 10, and 12 months of age. We started recruiting participants on June 11, 2022, and we anticipate completing data collection by September 2023. We are enlisting participants from Tehran primary healthcare clinics for the comparison group and from an obesity clinic for the exposure group.

Tehran primary healthcare clinics for the comparison group and the one of the obesity clinic for the exposure group.

#### Study setting

**Fig.1:**
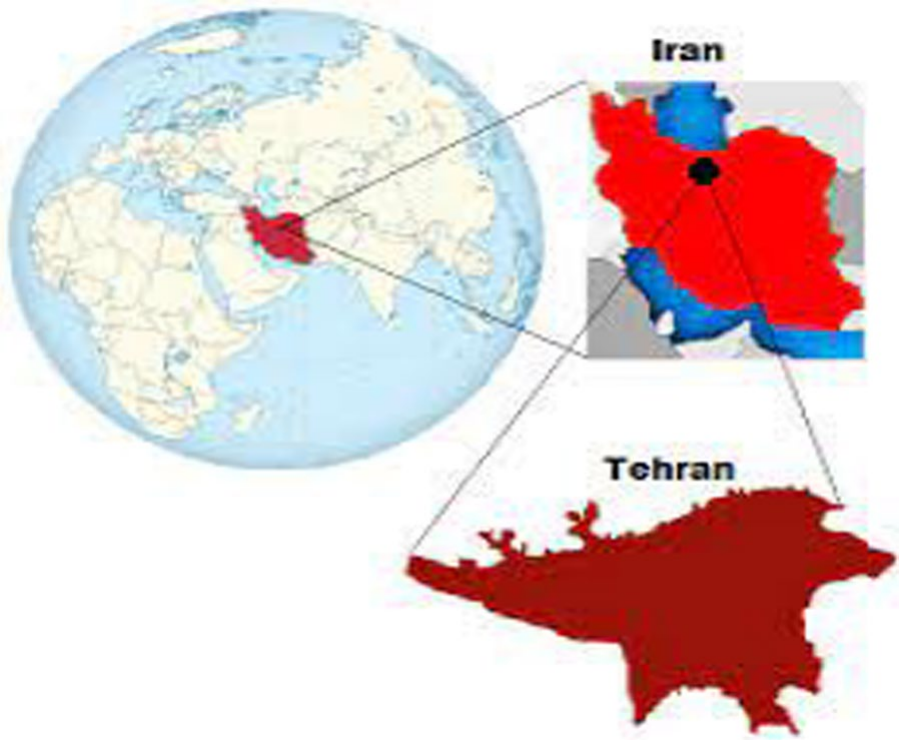
Schematic of the location of Tehran in Iran and the world

This study is taking place in Tehran, the capital of the Islamic Republic of Iran (Fig. 1). In this study, there are two environments for recruiting participants and completing questionnaires related to pregnancy:

1. Tehran primary healthcare clinics where pregnant mothers of the comparison group go to receive pregnancy care.
2. The one of the obesity clinic, and pregnant mothers in the exposure group have electronic surgical records in this clinic.

The information related to childbirth and some information post-natal period will be completed in the hospital where the delivery takes place, and other data after childbirth and the growth of the baby will be completed from the primary healthcare clinics where the women are covered.

### Study population

The population in this study will consist of two groups. The first group will include all pregnant women with a history of bariatric surgery who have an electronic file in the Obesity Clinic of Iran University of Medical Sciences (exposure group). The second group will include all pregnant women with obesity but without a history of bariatric surgery (comparison group) who are seeking prenatal care at selected primary healthcare clinics in Tehran and have a gestational age of less than 14 weeks and meet the eligibility criteria for the study.

#### Study sample

1. Pregnant women with a pregnancy of less than 14 weeks who have surgery records in the National Obesity Surgery Database (NOSD) in one of the Obesity Clinic in tehran.
2. Pregnant women with less than 14 weeks of pregnancy who have an electronic file in the Sina System (Integrated Health System) of primary healthcare clinics in Tehran.

#### Inclusion criteria

Consent to participate; have a gestational age below 14 weeks determined by either the first day of their last menstrual period (LMP) or first trimester ultrasound; be between the ages of 18–40; have no history of drug abuse (e.g. opium, heroin, methamphetamine); be experiencing their first pregnancy after bariatric surgery and not have undergone revisional bariatric surgery; have a body mass index (BMI) above 30 kg/m2 if they have obesity without a history of surgery; not have uncontrolled blood pressure (systolic blood pressure 160 mm Hg or diastolic blood pressure 100 mm Hg), recent history of cardiovascular disease (myocardial infarction or stroke in the last 6 months), hemoglobin A1C 11%, clinically significant liver or kidney disease, autoimmune diseases, or cancer; not have experienced severe stressful situations in the last 6 months such as the death of close relatives, domestic violence, accidents, or divorce; and not have a history of mental illnesses such as severe depression (scoring higher than 20 on the DASS-21 questionnaire), psychosis, or dementia diagnosed by a doctor and taking psychotherapeutic drugs such as lithium or amitriptyline.

#### Exclusion criteria

Terminate their pregnancy before 14 weeks (spontaneously or induced) or give birth before completing the third trimester questionnaires. These samples will be removed from analysis of variables such as micronutrients, stress, anxiety, and depression, but will still be investigated for pregnancy outcomes. Other exclusion criteria include reluctance to perpetuate participating in the study; failure to conduct necessary tests (especially those related to micronutrients), and inability to be followed up.

#### Sample size

With the assumption of a type 1 error of 5% and a type 2 error of 20%, and a comparison of the percentage of preeclampsia in two groups (one with bariatric surgery and one without), where the percentages are 23% and 5% respectively, a sample size of 68 individuals per group was estimated using the formula for comparing two ratios [13].

$${\text{n }} = \, \left( {{\text{Z}}_{{\alpha /{2}}} + {\text{Z}}_{\beta } } \right)^{{2}} * \, \left( {{\text{p}}_{{1}} \left( {{1} - {\text{p}}_{{1}} } \right) \, + {\text{p}}_{{2}} \left( {{1} - {\text{p}}_{{2}} } \right)} \right) \, / \, \left( {{\text{p}}_{{1}} - {\text{p}}_{{2}} } \right)^{{2}}$$

P_1_ =  23, P_2_ = 0.5

### Recruitment and consent procedures

The first step of project implementation: In the exposure group (pregnant women with a history of bariatric surgery): The first step involves extracting the list of names of married women with a history of obesity or morbid obesity and bariatric surgery from the National Obesity Surgery Database (NOSD) for the exposure group, which consists of pregnant women. The women will then be contacted via the telephone lines of Rasul Akram Hospital obesity clinic to confirm their pregnancy status and the age of their pregnancy. For those who are less than 14 weeks pregnant, the study objectives will be briefly explained, and they will be invited to participate in the research project voluntarily. The researcher's mobile number will be provided to them, allowing them to express their interest through SMS without any pressure or coercion. It is important to note that if any research participants drop out during the study, they will be replaced by another individual.

In the comparison group (obese pregnant women with a body mass index above 30): several primary healthcare clinics in Tehran will be chosen based on their BMI care registry offices to obtain over 30 samples. The maternal and child health unit of the selected centers will provide a list of pregnant women with mothers or Sina system (integrated health system) who are under 14 weeks of pregnancy, along with their names and phone numbers. These women will be contacted through messaging, phone calls, or social networks to explain the study's goals and ask if they are interested in participating.

If the women meet the study's entry criteria and express interest in participating, they will be asked to attend the obesity clinic on scheduled dates with a complete manual file (for more certainty) and all their previous tests (in the group with a history of surgery). The eligible pregnant women will be given an explanation of the study objectives, and informed written consent will be obtained before recruitment. Once consented, the participants will be registered into the cohort.

### Baseline assessment or second step of project implementation

In the face-to-face visit, will be conducted to measure the participants' height, weight, and body mass index. Additionally, pre-pregnancy BMI, pre-bariatric surgery BMI, and blood pressure will be checked. Ultrasound and routine pregnancy tests will also be aseessed to determine if the participant meets the inclusion criteria. If they do not meet the criteria, they will be excluded from the study. However, if they do meet the criteria, a Demographic-fertility information form will be completed. This form will gather information related to previous pregnancies, medical history, and bariatric surgery, including the type of operation and the time of receiving the surgery.

### Study tools

We will be utilizing various tools to evaluate the intended measures, which are outlined below:

1. A researcher-made questionnaire to evaluate maternal and fetal/newborn outcomes: This includes surgical details such as the type of surgery and the duration since the surgery, individual infertility information such as the woman's age, education level and employment status of both partners, and length of cohabitation. The questionnaire also covers infertility history and information related to maternal and fetal/newborn outcomes during pregnancy, childbirth, and the neonatal period. Additionally, it records the results of prenatal tests and micronutrient levels. The questionnaire has been designed by gynecologists, obstetricians, a nutritionist, and an experienced bariatric surgeon. Its face validity has been confirmed by 10 obstetricians and gynecologists in Tehran.
2. 24-h food recall: One way to assess a person's nutritional status is through the 24-h food recall method, where the individual is asked to recall and report all food and drink consumed within the past 24 h. In this study, pregnant women will receive a reminder form and instructions via messaging apps like WhatsApp or Telegram. They will be asked to record all food and drink consumed during three days of each trimester of pregnancy (two non-holiday days and one holiday weekday) on a provided form and submit a photo of it.
3. Physical Activity Questionnaire in Pregnancy (PPAQ): The Physical Activity Questionnaire is a validated tool for measuring physical activity during pregnancy [68]. It consists of two parts: personal characteristics and 32 questions about activities at home, commuting, sports, and work. The intensity of physical activity is calculated using Met, which estimates metabolic cost. Sedentary activity has a Met less than 1.5, light activity has a Met of 1.5–3, moderate activity has a Met of 3–6, and vigorous activity has a Met greater than 6 [69]. The questionnaire is completed through a telephone interview in the first and third trimester (between 32–36 weeks of pregnancy) of pregnancy. The Persian version of the questionnaire has good reliability, with an ICC coefficient of 0.87 and a Cronbach's alpha coefficient of 1.69 [70]. In this study, the questionnaire's reliability will be assessed using Cronbach's alpha.
4. Stress, anxiety and depression questionnaire (DASS-21): The DASS-21 questionnaire is used to assess negative emotional states such as depression, anxiety, and stress. It consists of 21 questions, with 7 questions for each category. The person completing the questionnaire must indicate the status of a symptom during the last week using a 4-point Likert scale. Scores range from 0 to 21, with scores above 14, 10, and 17 indicating severe depression, anxiety, and stress, respectively. Mild depression, anxiety, and stress are indicated by scores of 4–5, 6–8, and 5–6, respectively. The questionnaire will be completed via telephone interview during the first and third trimester (between 32–36 weeks of pregnancy) of pregnancy. The internal consistency coefficients (Cronbach's alpha) for depression, anxiety, and stress subscales are 0.88, 0.82, and 0.90, respectively [71]. The reliability of the questionnaire will be examined through internal consistency in this study.

### Data collection

**Fig. 2:**
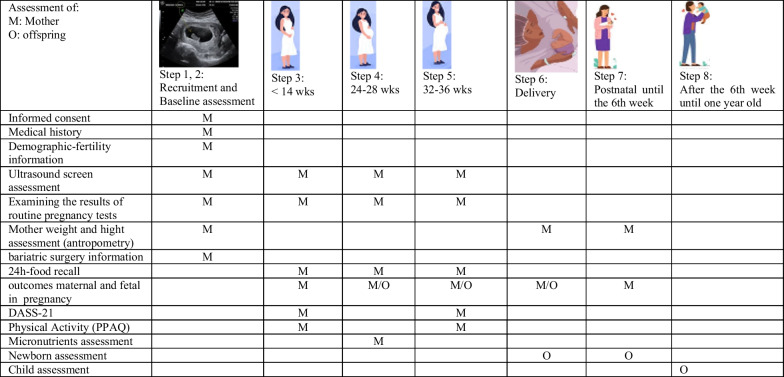
Data collection schedule per steps during this study. Prenatal data collection time points are at < 14 weeks’ gestation (step 1, 2 and 3); 24–28 weeks (phase 4); 32–36 weeks (phase 5) and at delivery (phase 6). Postnatal data collection time points until the 6th week (phase 7); after the 6th week until one year old (2, 4, 6, 8, 10 and 12 months of age) (phase 8)

In Fig. 2, an overview of the data collection process is presented, including the seven step and eight time points (referred to as "steps") across the project's duration. Two steps have already been explained, and the remaining six steps are outlined below:

### The third step of project implementation (completion of questionnaires related to the first trimester of pregnancy)

Due to the high volume of questionnaires and the possibility of participants experiencing nausea, vomiting, or other pregnancy-related symptoms, physical activity and DASS-21 questionnaires will be completed via telephone interview when the mother is feeling well. Participants will also be asked to complete and send a 24-h feeding reminder as soon as possible. During the interview, questions will be asked about pregnancy outcomes in the first trimester, including nausea and vomiting, miscarriage, vaginal bleeding, ectopic pregnancy, and molar pregnancy. Additionally, pregnant women will be requested to send the results of screening tests and ultrasound via WhatsApp or Telegram.

The criteria for matching include the mother's age (± 2 years), parity (primiparous or multiparous), pre-surgery BMI category (using early-pregnancy BMI in controls; 30 to < 35, 35 to < 40, 40 to < 45, 45 to < 50 or _50 kg/m^2^) and educational level (Elementary school/ Middle school/ High school/ college).

### The fourth step of project implementation (collecting the information needed in the second trimester of pregnancy)

During the second trimester, will be assessed and document any consequences for both the mother and fetus, such as pregnancy-related illnesses like severe nausea and vomiting, pre-eclampsia, gestational diabetes (GDM), abortion, premature birth, placenta previa and placental abruption, venous thrombosis, and fetal abnormalities. Additionally, participants are asked to complete and submit a food diary for three days of the second trimester. Then, If desired, participants may provide their national code to record tests related to micronutrients (zinc, calcium, magnesium, selenium, copper, vitamins A, B9, B12, 25-hydroxy Vit D), ferritin, albumin, glucose tolerance test OGTT (in the group exposed to sugar one and two hours after a meal to prevent dumping syndrome) and hemoglobin A1C The general physician of the obesity clinic will administer these tests, but if participants do not wish to provide their national code, we will send them a list of tests via SMS. We will ask participants to perform these tests (after 8–12 h of fasting) at 24–28 weeks of pregnancy under the supervision of their gynecologists. We will also teach pregnant women how to perform these tests and provide them with a choice of five laboratories (located in the center, north, south, east, and west of Tehran) to conduct the tests. As these tests are necessary for the exposure group but not for the comparison group, we will cover the cost of the micronutrient tests to avoid additional expenses for the comparison group.

*Note: Between 24 and 28 weeks of gestation, a 2-h 75g oral glucose tolerance test (OGTT) is conducted using standard procedures to determine the development of gestational diabetes mellitus (75). However, there are no validated standards for the required levels of vitamins and trace elements during pregnancy. The values for micronutrient deficiencies, which are defined as serum trace element levels below the lower reference level, are based on usual observed values in generally healthy pregnant women during the second trimester of pregnancy. This information is derived from a comprehensive literature review and recent Iranian clinical practice guidelines for pregnancy after bariatric surgery. To summarize, standard procedures are used for the OGTT, but there are no validated standards for micronutrient levels during pregnancy.

**The fifth step of project implementation (collecting the information needed in the third trimester of pregnancy):** During this stage, will be reviewed all fetal and maternal outcomes related to pregnancy, including pre-eclampsia, diabetes, premature and post-term delivery, anemia, oligohydramnios, polyhydramnios, premature rupture of the amniotic sac, uterine growth restriction, venous thrombosis, gestational weight gain (GWG), GDM, perinatal mortality (stillbirth or neonatal death after 20 weeks gestation), pregnancy-induced hypertension/pre-eclampsia, and vaginal bleeding. Additionally, pregnant women will complete the DASS-21 AND PPAQ questionnaires again through a telephone interview, and the research units will complete the food reminder form again in the third trimester. Participants are asked to perform routine tests related to 28–32 weeks of pregnancy, such as a CBC to check for anemia and a urinalysis to check for proteinuria and urinary infection. Throughout the pregnancy, we will ask the mother about any trauma to herself or the fetus, as well as any hospitalizations and the reasons for them. Also, pregnant women from both groups will send the results of tests such as micronutrients, ferritin, and albumin to the researcher, who will then send them to the project nutritionist. The nutritionist will provide recommendations for vitamins and minerals based on any deficiencies identified in the tests, and pregnant women will be asked to take the supplements under the supervision and approval of their gynecologist.

**The sixth step of project implementation (gathering the required information during delivery):** At the end of the pregnancy, the research units are required to inform the researcher about the expected time of delivery. On the day of delivery, various intrapartum variables will be recorded, including the date and time of birth, gender, changes in the mother's weight during pregnancy, type of labor (spontaneous, induced, or planned cesarean delivery), mode of delivery (vaginal or cesarean), type of anesthesia, induction and failure of labor, shoulder dystocia, placental abruption, presence of meconium-stained liquor, and maternal outcomes such as postpartum hemorrhage, perineal tear, episiotomy, hysterectomy, and neonatal outcomes including 1 and 5-min Apgar score, preterm delivery, need for resuscitation or intubation, admission to the neonatal intensive care unit, neonatal death, height, head circumference, and birth weight for examination of macrosomia, low birth weight, small or large for gestational age, and intrauterine growth restriction. The information will be collected by referring to the birth record in the hospital where the participant gives birth, including malformations, respiratory distress, stillbirths, and neonatal mortality.

**The seventh step of project implementation (gathering the required information after delivery): **At this stage, information related to the condition of the mother and the fetus, includes any bleeding that occurred after delivery and how it was treated, hypertension, infections at the episiotomy or caesarean site, venous thrombosis, Apgar score at 5 min, the infant's feeding methods and any breastfeeding problems, the mother's weight 6 weeks after delivery, the baby's weight at 28 days, and the results of foot tests. This information is obtained through phone interviews with the participants, as well as from the birth record at the hospital and the mother's electronic record in the Sina system.

The eighth step of project implementation (collecting information related to the child’s growth up to one year old): At this stage, will be recorded child's weight, height and head circumference every 2 months until they reach one year of age. These values can be tracked and reviewed using the SINA system or the child’s vaccine card.

## Some diagnostic criteria in this study

Gestational age at delivery: “measuring the craniocaudal length during a first-trimester ultrasound or by using the date of the last menstrual period”. Pre-term delivery: “gestational age at delivery is less than 37 weeks”, prolonged pregnancy: “gestational age is 41 weeks or more”. Postpartum hemorrhage (PPH): “blood loss of 1000 mL or more” [72]. Small for gestational age fetus (SGA): “ultrasonographic estimated fetal weight below the 10th percentile for gestational age”, Large for gestational age fetus (LGA): “fetal weight above the 90th percentile”. Macrosomia: “baby's birthweight over 4000 g”, Intrauterine growth restriction:“fetal growth restriction observed on ultrasound examination” [73]. Pregnancy-induced hypertension: “hypertension above 140/90 mmHg at two occasions without proteinuria after 20 weeks of gestation in a previously normotensive woman” [74].

Preeclampsia: “systolic pressure ≥140 mmHg and/or diastolic pressure ≥90 mmHg, occurring after 20 weeks of pregnancy in women who were previously normotensive, accompanied by specific levels of proteinuria”. Gestational diabetes: “First trimester: glucose ≥ 92 mg/dL and ≤126 mg/dL. Second trimester: overload of 75 g of glucose glycemia ≥ 92 mg/dL for fasting and/or 180 mg/dL for 60 min after overload and/or 153 mg/dL for 120 min after overload", Anemia: "hemoglobin level ≤11 g/dL”, Premature births: “Gestational age at birth less than 37 weeks”, Respiratory distress: “Need for masked breathing cycles and Amsterdam Medical Breath Unit (AMBU), Continuous Positive Airway Pressure (CPAP) or tracheal gold intubation”, Fetal malformation: “Any fetal anatomical change” and Neonatal ICU: “stay covers any length of stay in the neonatal ICU, even if it is less than 24 hours” [74–78].

## Data management and analysis

### Data management

Raw data is saved in password-protected Microsoft Access documents, accessible only to the data input operator and principal investigators. Pregnant women's information is anonymized and linked to separate documents. The captured data is checked for errors by the researcher, and the final version is stored in protected zipped files. In order to calculate the amount of calories, fat, carbohydrates and protein received through the consumed diet, a print is taken from the food note forms that are completed by pregnant women and sent to the dietitian. Then Dietary data by dietitian are entry in Microsoft Excel (Microsoft Corporation, Washington, USA) and all electronic entries are double checked for the correct food code and a reasonable amount reported. Finally, the amount of energy and macronutrients received by the participants will be measured by Nutritionist IV nutritional software. The data related to the physical activity of pregnant women are calculated twice by the researcher and added to other data. Also, the data related to stress, anxiety and depression of pregnant women are reviewed and graded according to the instructions for calculating the score in the DASS-21 questionnaire. The final dataset of pregnant women will migrate into the Software for Statistics and Data Science (SPSS Inc., version 22.0, Chicago, IL, USA) for analysis.

### Data analysis

We will describe continuous variables using mean ± SD and compare them between groups using Student’s t-tests (or Mann–Whitney U test if appropriate). Categorical variables will be described using percentages and compared between groups using chi-squared test (or Fisher's exact test if appropriate). We will use Spearman's correlations to describe the associations between serum micronutrient values in the second trimester and neonatal and maternal outcomes. We will adjust the data for potential time-dependent and static covariates and use logistic and linear regression models to control for confounders. The level of significance will be set at P < 0.05.

## Discussion

In June 2022, we conducted a face validity test on our researcher-made questionnaire. Then, Based on expert feedback, changes were done to improve the quality of data and sample collection procedures. Then, added several data fields to the questionnaires to ensure the examination of all maternal and fetal/neonatal outcomes. In the next step, investigated the reliability of tools including DASS-21 and Physical Activity Questionnaire in Pregnancy. To make it easier to complete the food note form, we prepared a short instruction with the help of a nutritionist (our project partner). After receiving the code of ethics, we started sampling and are currently in the follow-up phase to check outcomes after childbirth.

The advantages of this study include the following:

First, it's a prospective cohort study, which means it can provide valuable insights for future research. Second, by analyzing micronutrient levels in the blood of pregnant women who have undergone bariatric surgery, we can better understand the impact of the procedure on the health of both the mother and the fetus/newborn. This knowledge can help specialists and patients make more informed decisions about the surgery and its potential outcomes. Additionally, if any adverse changes occur after the surgery, targeted measures can be taken to address them. The study also sheds light on issues that haven't been explored before, such as stress, anxiety, and depression in women who have undergone bariatric surgery in Iran. By recognizing these problems, we can better understand how they affect pregnancy outcomes and develop appropriate interventions. One of the practical goals of this study is to help develop guidelines for pregnancy supplements in pregnant women with obesity, with and without a history of bariatric surgery. By using the results of this study, we can better meet the needs of patients in the future.

## Data Availability

Data sharing is not applicable to this article as no datasets were generated or analysed for the purposes of this protocol article.
